# Predictable Molecular Adaptation of Coevolving *Enterococcus faecium* and Lytic Phage EfV12-phi1

**DOI:** 10.3389/fmicb.2018.03192

**Published:** 2019-01-31

**Authors:** Stephen Wandro, Andrew Oliver, Tara Gallagher, Claudia Weihe, Whitney England, Jennifer B. H. Martiny, Katrine Whiteson

**Affiliations:** ^1^Department of Molecular Biology and Biochemistry, University of California, Irvine, Irvine, CA, United States; ^2^Department of Ecology and Evolutionary Biology, University of California, Irvine, Irvine, CA, United States; ^3^Department of Pharmaceutical Sciences, University of California, Irvine, Irvine, CA, United States

**Keywords:** phage (bacteriophage), *Enterococcus*, experimental evolution, phage therapy, tail fiber, exopolysaccharide, coevolution

## Abstract

Bacteriophages are highly abundant in human microbiota where they coevolve with resident bacteria. Phage predation can drive the evolution of bacterial resistance, which can then drive reciprocal evolution in the phage to overcome that resistance. Such coevolutionary dynamics have not been extensively studied in human gut bacteria, and are of particular interest for both understanding and eventually manipulating the human gut microbiome. We performed experimental evolution of an *Enterococcus faecium* isolate from healthy human stool in the absence and presence of a single infecting Myoviridae bacteriophage, EfV12-phi1. Four replicates of *E. faecium* and phage were grown with twice daily serial transfers for 8 days. Genome sequencing revealed that *E. faecium* evolved resistance to phage through mutations in the *yqwD2* gene involved in exopolysaccharide biogenesis and export, and the *rpoC* gene which encodes the RNA polymerase β’ subunit. In response to bacterial resistance, phage EfV12-phi1 evolved varying numbers of 1.8 kb tandem duplications within a putative tail fiber gene. Host range assays indicated that coevolution of this phage-host pair resulted in arms race dynamics in which bacterial resistance and phage infectivity increased over time. Tracking mutations from population sequencing of experimental coevolution can quickly illuminate phage entry points along with resistance strategies in both phage and host – critical information for using phage to manipulate microbial communities.

## Introduction

Bacteriophages (phages) drive microbial diversity and function at both broad (Bouvier and Giorgio, 2007) and fine scales (Mcshan et al., 2016) through their influences on bacterial community composition (Stern et al., 2012) and bacterial pathogenesis (Davis et al., 2000). Phages are estimated to be present at 10^9^ virions per gram in the gut (Kim et al., 2011) and are therefore likely to have major influences on beneficial and pathogenic gut bacteria. Phages that lyse their host (lytic phages) or alter host virulence gene expression (some temperate phages) present a potentially rich pool of new therapies against antibiotic resistant pathogens (Wright et al., 2009; Oechslin et al., 2016). Recently, the clinical application of phages against highly antibiotic resistant bacteria (Viertel et al., 2014; Chan et al., 2016; Schooley et al., 2017) has highlighted the need for well-controlled experiments that investigate the molecular interactions between phage and bacteria. Before phage-based therapies can be developed, we must have a solid understanding of how a targeted bacterial pathogen may evolve resistance to a treatment phage, and how the treatment phage responds to host resistance.

Reciprocal evolution of bacteria and phage, or coevolution (Thompson, 1999), has been well-studied (Koskella and Brockhurst, 2014; Martiny et al., 2014; Scanlan, 2017) in two model systems: *Pseudomonas fluorescens* and *Escherichia coli* (Bohannan and Lenski, 2000; Hall et al., 2011; Koskella and Brockhurst, 2014). Although we can learn broad principles from these model systems, their study cannot replace experiments with more clinically relevant organisms to understand human associated phage-bacterial interactions. We aimed to investigate coevolution in *Enterococcus faecium*, a common, but low-abundance member of the human gut microbiome that is also an important opportunistic pathogen. The World Health Organization classifies vancomycin-resistant *E. faecium* as a Priority 2 level pathogen in need of new antibiotic therapies (Lawe-Davies and Bennett, 2017). Common enterococci infections include endocarditis, blood/wound infections, and urinary tract infections (Koch et al., 2004). Enterococci can also become dominating members of the gut community following antibiotic perturbation (Hendrickx et al., 2015), leading to dysbiosis and increased likelihood of infection (Van Tyne and Gilmore, 2014). Developing a coevolution model using lytic phage and *Enterococcus* could therefore be a useful step toward addressing this global health threat. Coevolution experiments can quickly reveal candidates for the molecular basis of *Enterococcus*-phage interactions so that optimal cocktails of phages can be constructed. Indeed, cocktails of multiple phages with orthogonal infection mechanisms hold great promise as therapeutics (Yen et al., 2017; Nale et al., 2018).

The evolution of resistance to phage infection has been well documented and can happen through many routes. These include blocking phage adsorption through mutation, restriction-modification systems, CRISPR-Cas systems, and abortive infection (Dy et al., 2014). In addition, new mechanisms of phage resistance are still being discovered (Doron et al., 2018), which highlights the potential for discovery in the interactions between bacteria and phages. Coevolution between *Enterococcus* and its phages remains poorly studied, but resistance to one *Enterococcus* phage has been shown to evolve through mutation of an integral membrane protein to prevent phage adsorption (Duerkop et al., 2016). This remains one example, and *Enterococcus* may utilize an entirely different resistance mechanism during coevolution with a different phage.

We experimentally coevolved *E. faecium* with a lytic phage (EfV12-phi1) to characterize the genomic and phenotypic outcomes of their interaction. Phage EfV12-phi1 was isolated from sewage and has been previously referred to as “1” or “Φ1” (Jarvis et al., 1993). It is a member of the Twort-like family of Myoviridae phages, a group of strictly lytic phages that infect Firmicutes and generally demonstrate a broad host range. Closely related Twort-like phages have been previously employed for phage therapy and have demonstrated lethality against a long list of clinically relevant bacterial strains, including vancomycin-resistant enterococci (VRE); Group B, C, E, G *Streptococcus*; *Staphylococcus aureus*, and others (Klumpp et al., 2010; Khalifa et al., 2015, 2018). The lysin of phage EfV12-phi1 has been previously shown to kill species of *Enterococcus* (including VRE), *Streptococcus*, and *Staphylococcus* (Yoong et al., 2004).

We conducted four coevolution experiments where phage EfV12-phi1 was grown with *E. faecium* with 1:10 serial transfers twice daily, so that a large fraction of the population is carried over. To differentiate between genomic changes associated with coevolution versus those that might be due to laboratory adaptation, we compared these experiments to parallel control experiments where *E. faecium* was grown alone or phage EfV12-phi1 was propagated on a naïve host. Based on phage-host experiments in model systems, we expected to see mutations arise in the phage tail fibers that allow phage to recognize and bind their hosts and in bacterial surface receptors where phage often enter their hosts (Silva et al., 2016).

## Materials and Methods

### Bacterial Strains and Phage

The bacteria used in this study was *Enterococcus faecium* strain TX1330 (BEI HM-204), was isolated from healthy human feces and obtained through BEI Resources, NIAID, NIH as part of the Human Microbiome Project. The phage used for this study was *Enterococcus* Phage EfV12-phi1, isolated on *Enterococcus faecalis*, from Canadian sewage in 1975 (HER number 339; d’Herelle collection, Laval University, Quebec, QC, Canada).

### Coevolution of *Enterococcus* Bacteria and Phage

A culture of *E. faecium* TX1330 growing exponentially (OD600 = 0.3) in brain heart infusion (BHI) broth was split into twelve replicates of 10 mL culture in 15 mL Falcon tubes. Four replicates were designated bacterial host control, four were phage control, and four were coevolution. Phage EfV12-phi1 was added to the coevolution and phage control cultures at an MOI of approximately 0.003. Cultures were incubated shaking with loose caps at 37°C. Every 12 h for 8 days, 1 mL of the replicate host control and coevolution tubes were inoculated into 9 mL of new BHI broth. For the phage control, 1 mL of phages was separated from bacteria by syringe filtration through a 0.2 um polyethersulfone filter (GE Healthcare Life Sciences) and mixed with 1 mL of the contemporary host control in 8 mL of new BHI broth. Performing 1:10 dilutions at each passage, we estimate 3.5 generations (doublings) are required to reach stationary phase again, resulting in approximately 56 generations total. After each dilution, 900 uL of 12-h culture containing the population of bacteria and phages was added to 600 uL of 50% glycerol and stored at -80°C. Transfer numbers 1, 4, 8, 12, and 16 were chosen for sequencing. At each timepoint (after 12 h of growth), the OD600 of each culture was measured.

### Host Range Assay

Host range of phage and bacterial isolates from *E. faecium* were determined with streak assays as described before (Harcombe and Bull, 2005). Bacteria and phage were isolated from the first and last timepoints of the host control and coevolution replicate populations one and two. Bacteria were isolated by streak plating and picking single colonies. Phages were isolated by performing a double agar overlay with 100 uL of the raw population and then picking plaques (so that phages are growing on contemporary hosts from the same replicate population). Phages were amplified by performing plaque assays on ancestral hosts and harvested by soaking plates in 5 mL SM buffer followed by filtration of collected SM buffer through a 0.2 um syringe filter. 20 uL of each bacterial isolate and 20 uL of each phage isolate was streaked perpendicularly across an agar plate. The intersection of the bacteria and phage was examined and scored for lysis. In total, three ancestral hosts and 12 coevolved hosts were crossed against six ancestral phages and 16 coevolved phages.

### DNA Extraction, Library Preparation, and Sequencing

DNA was extracted from the populations of bacteria and phages in the chosen timepoints with the Zymo Universal DNA extraction kit using the recommended protocol provided by the manufacturer. Sequencing libraries were prepared with Illumina’s Nextera kit using methods outlined in Baym et al. (2015). The libraries were loaded onto an Illumina Next-Seq at 1.8 picomolar concentration using Illumina’s mid-output kit for 75 bp paired end sequencing.

A more complete bacterial reference genome was assembled using Oxford Nanopore’s 1D Genomic DNA Ligation kit (Goodwin et al., 2017). Briefly, DNA was repaired using the FFPE DNA repair kit (New England Biolabs) and cleaned up using AMPure XP beads (Beckman Coulter). The repaired DNA was dA-tailed using NEBNext Ultra End Repair (New England Biolabs) and sequence adapters were ligated using Blunt TA ligase master mix (New England Biolabs). The MinION sequencer was primed, per manufacturer’s instructions, and 700 ng of DNA was loaded onto the sequencer. The run was allowed to generate data for 48 h. Sequence data from the MinION and Illumina sequence data from timepoint one of the host control were used together to generate a host reference genome using the MIRA assembler (Chevreux et al., 1999).

### Genome Assembly and Annotation

The reference genome for *E. faecium* TX1330 was assembled using reads from time point 1 of the host control. Reads were assembled using the PATRIC smart assembler (Wattam et al., 2017), which combines the two best assemblies from SPAdes (Bankevich et al., 2012), IDBA (Peng et al., 2010), and Velvet (Zerbino and Birney, 2008) assemblers. The phage was assembled using SPAdes (Bankevich et al., 2012). The resulting contigs were annotated using PATRIC’s annotation pipeline, which uses RASTtk for gene calls (Wattam et al., 2017). The sequenced genome of *E. faecium* TX1330 can be found at GenBank: GCA_003583905.1, and the EfV12-phi1 genome can be found at GenBank: MH880817.

### Genomic Mutation Analysis

Paired-end reads were run through Breseq (Deatherage and Barrick, 2014) once using the ancestral phage EfV12-phi1 as the reference genome and once using the ancestral *E. faecium* TX1330 with default parameters. Briefly, Breseq uses Bowtie2 (Langmead and Salzberg, 2012) to align reads to a reference genome and creates a SAM file which SAMtools converts to a pileup file. Custom R scripts were then used to parse through the resulting alignments and detect mutations at greater than 10% frequency. Mutations were labeled as synonymous or non-synonymous by Breseq, and all predicted non-synonymous mutations were manually investigated using Geneious (Biomatters v9.0). All bacterial mutations were visualized using Geneious and phage mutations using Geneious and ggplot2 in R.

### Tail Fiber PCR

Phage populations from the final timepoint of all four replicates were grown by adding 10 uL of the frozen timepoint 16 cultures to 10 mL BHI and grown overnight shaking at 37°C. Cultures were then spun down and the supernatant was syringe filtered through a 0.2 um polyethersulfone filter. Phages were then concentrated down to 1 mL using Amicon 100 kDa centrifugal filter units. DNA was extracted from concentrated phages using Zymo Universal DNA extraction kit.

Primers were designed outside the duplicated region of the tail fiber gene so that the amplicon would be longer if the region was duplicated. The primers used were F: 5′ TGTTGCACCAGAAAACGCAG 3′ and R: 5′ AGGTCTGTACGAGCCGTGTA 3′. PCR was run using Phusion polymerase with the following protocol: 98°C: 30 s (98°C 10 s, 53°C 30 s, 72°C 10 min) x 35, 72°C 10 min. Amplicons were visualized on a 1% agarose gel using Invitrogen SYBR gel stain.

### Location of Mutations in RNA Polymerase B’ Structure

The structure of the *E. coli* RNA polymerase B’ subunit was downloaded from Protein Data Bank (4JK1, DOI: 10.2210/pdb4JK1/pdb). The amino acid sequence of the *E. faecium* TX1330 RNA polymerase B’ subunit was aligned to the *E. coli* sequence to find the corresponding locations. The structure and locations of mutations were visualized using PyMOL (Schrödinger, 2015).

### MinION Sequencing of Tail Fiber Duplication

The Oxford Nanopore MinION sequencer was used to sequence a phage isolate that contained the tail fiber duplication. The phage was isolated by picking a plaque from timepoint 16 of replicate 4 (directly plating 100 uL of the population). The phage isolate was propagated on a contemporary (final timepoint) *Enterococcus* isolate from population 4 to get enough phage DNA for sequencing. DNA was extracted using a Zymo Quick-DNA micro kit. DNA was prepared for MinION sequencing according to manufacturer’s recommendations using the 1D Genomic DNA by ligation protocol as described above. A total of 199,734 reads were generated with a median sequence length of 3,057 bp. Bowtie2 was used to extract the 57% of reads that aligned to the phage genome; the remaining reads aligning to the bacterial genome were discarded. The data was analyzed in Geneious to determine the number of duplications in the tail fiber gene. A total of 5,400 reads aligned to the tail fiber gene and were over 3 kb so could span the length of a single duplication (1.8 kb).

## Results

### *E. faecium* and Phage EfV12-phi1 Display Arms-Race Coevolution Dynamics

**FIGURE 1:**
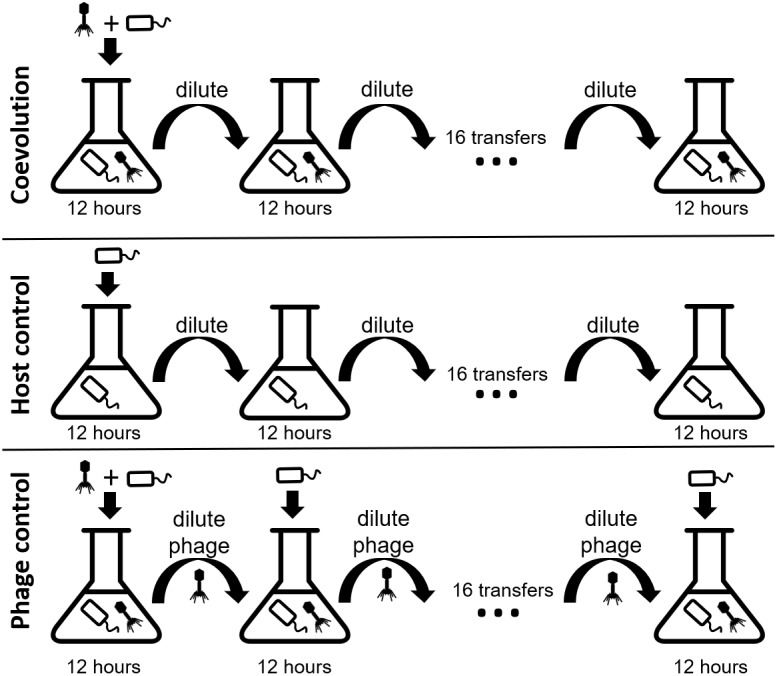
Experimental design of the three branches of the study. In each branch, phage and bacteria or only bacteria were added to a microcosm and allowed to grow for 12 h before being diluted 10-fold. The phage control filtered out the bacteria during each dilution, preventing bacterial coevolution. Each branch was done in quadruplicate. Icon credit: thenounproject.com.

**FIGURE 2:**
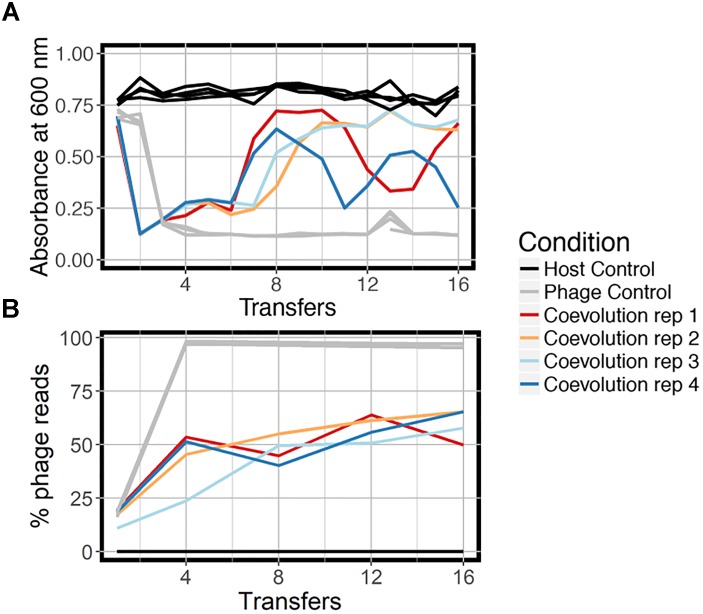
Growth dynamics of experimental coevolution. **(A)** Optical density of the bacteria for each branch of the experiment, measured at the end of 12 h prior to diluting back 10-fold in fresh BHI media. All replicates of the host control and phage control are shown in the same color because there was little variation. **(B)** Proportion of total sequenced reads mapping to phage EfV12-phi1 indicates the relative abundance of this phage at each timepoint. Reads that did not map to phage mapped to *E. faecium*.

We coevolved *E. faecium* with lytic phage EfV12-phi1 as four replicate microcosms, passaging 16 times in 8 days (every 12 h), allowing for approximately 53 generations (Figure 1). Bacterial host control cultures were also set up in quadruplicate with identical conditions minus the phage. Quadruplicate phage controls were established by growing the phage on a naïve host, separating the phage from the host during each passage, and then adding the phage lysate to an independent aliquot of the naïve host control culture. Bacterial growth was monitored daily by optical density readings, which decrease when bacterial cells are lysed by phage. Phage infection initially reduced the density of all four bacterial cultures during the first day. This was followed by increased optical density after six to seven transfers (depending on the replicate), indicative of the evolution of resistance to phage (Figure 2A). In two replicate cultures, optical densities did not decline again after initial resistance arose, whereas in the other two replicate cultures, optical densities oscillated for the duration of the experiment.

At the final timepoint, bacterial populations in three of four replicates remained at a high optical density, despite relatively high concentrations of phage DNA (an approximation of phage abundance; Figure 2B). As expected, optical densities in the phage control cultures with naïve bacteria were consistently reduced upon infection by EfV12-phi1, and host control cultures (with no infecting phage) showed no reductions in optical density.

**FIGURE 3:**
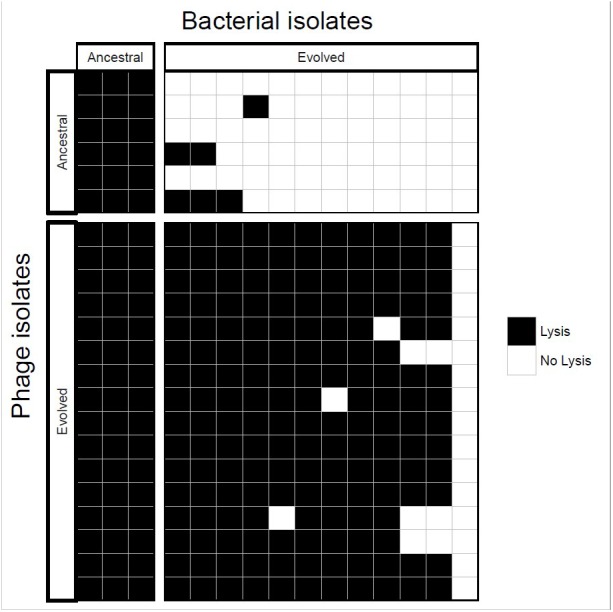
Host range analysis of phage and bacterial isolates. Bacteria and phage isolated from the initial and final timepoints were tested for infectivity. Each box represents whether lysis occurred when a single phage isolate crossed with a single bacterial isolate.

Ancestral and coevolved bacterial isolates were challenged with infection by ancestral and coevolved phages and bacterial lysis was scored using a plate-based assay (see Methods). These experiments showed that coevolved bacterial isolates (from the final coevolution timepoint) were resistant to ancestral phage isolates, and the coevolved phage isolates infected ancestral bacterial isolates (Figure 3). In most cases, coevolved phages infected coevolved bacteria, suggesting that at least one round of coevolution had occurred (*E. faecium* evolved resistance, EfV12-phi1 in turn evolved an expanded host range to overcome this resistance). These results are consistent with arms race coevolutionary dynamics in which bacterial resistance and phage infectivity increase over time.

### Resistance to Phage Evolves Though Exopolysaccharide and RNA Polymerase Mutations

To identify the bacterial mutations that led to resistance, and the phage mutations that enable infection of the freshly evolved host, we sequenced the populations from replicate microcosms at five timepoints (1, 4, 8, 12, and 16 transfers) from the coevolution treatment, three phage control timepoints (1, 4, and 16) and two host control timepoints (1 and 16). These population reads were mapped to the ancestral *E. faecium* genome that was sequenced by both Illumina NextSeq and Oxford Nanopore MinION, yielding a high-quality reference genome in three contigs and one plasmid contig. Mutation frequencies for the population were calculated based on the percentage of reads supporting the mutant base divided by the total coverage. Non-synonymous mutations were not observed in host control bacteria but were observed in seven genes in coevolving populations. Many of these genes encode hydrolases and transferases (Table 1). Two genes were mutated in all four replicates: putative tyrosine kinase *yqwD2* and RNA polymerase B’ subunit *rpoC*.

**Table 1:** All mutations present in *E. faecium* TX1330 at the final timepoint.

Replicate	Gene/predicted function	Type	AA change	Frequency (%)
1	*RpoC*	Non-synonymous snp	H419R	38.2
1	*RpoC*	Non-synonymous snp	S926T	21.1
1	*RpoC*	Non-synonymous snp	L800V	14.8
1	Hypothetical protein in capsule synthesis locus	Non-synonymous snp	M29I	30
1	*yqwD2*	Non-synonymous snp	P58L	37.6
1	*yqwD2*	Non-synonymous snp	P58H	19.2
2	Malonate decarboxylase beta subunit/malonate decarboxylase gamma subunit CDS	Non-synonymous snp	G148V	45.5
2	Predicted hydrolase of the HAD superfamily CDS	Nonsense	S191stop	33.3
2	murA – UDP-N-acetylglucosamine 1-carboxyvinyltransferase	Non-synonymous snp	G20C	40
2	*yqwD2*	Non-synonymous snp	P58H	100
3	*RpoC*	Non-synonymous snp	H419R	79.2
3	*yqwD2*	Non-synonymous snp	K89N	72.2
3	hydrolase, haloacid dehalogenase-like family CDS	Nonsense	E68stop	50
4	*yqwD2*	Non-synonymous snp	P58H	92.5

*All bacterial mutations are from timepoint 16 of coevolution replicates. No mutations were seen in the host controls. Locus tags for each gene can be found in Supplementary Table S1.*

**FIGURE 4:**
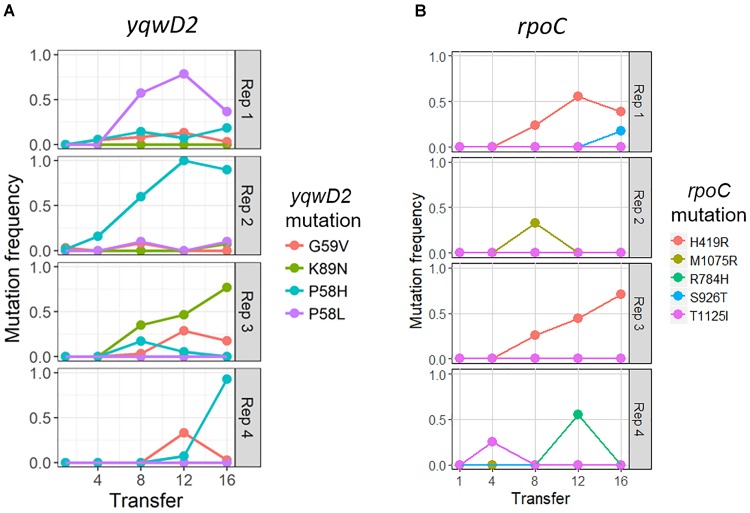
Frequency of common bacterial mutations over time. Population frequency of mutations in **(A)** capsule biosynthesis tyrosine protein kinase *yqwD2* and **(B)** RNA polymerase B’ subunit gene *rpoC*. All mutations present at a frequency of 10% in one timepoint in one replicate shown.

The putative tyrosine kinase, *yqwD2*, is involved in capsule exopolysaccharide production (Figure 4A). Replicates had different non-synonymous mutations within this gene: three occurred on neighboring amino acid residues (P58H, P58L, and G59V), while the fourth occurred twenty residues away (K89H) (Figure 4A). Mutations in the *yqwD2* gene were first detected at timepoint eight and became more frequent in the coevolving bacterial populations over time. The increasing frequencies of different mutations in the same gene suggest convergent evolution toward a single mechanism for resisting phage infection.

The second bacterial gene observed to mutate when coevolving with phage EfV12-phi1 was the *rpoC* gene encoding RNA polymerase β’ subunit (Figure 4B). A total of five different non-synonymous mutations were observed at high frequency in one or more replicates. The positions of mutations were mapped to the 3D structure of *E. coli’s* RpoC, showing that all five mutations are located near each other on the interior portion of the protein near the active site (Supplementary Figure S1).

### Phage EfV12-phi1 Combats Resistance Through Tandem Tail Fiber Duplications

Mutations in the phage genome were also tracked over time as the phage coevolved with the host bacteria. Four phage genes mutated throughout the experiment. Three of these mutations also occurred in all replicates of the phage controls, indicating that they are likely to generally increase infectivity for this specific host and are not a response to the evolution of bacterial resistance. One of the phage-control mutations occurred in a putative structural capsid gene and resulted in a change from asparagine to lysine. In all replicates, this mutation started at a low frequency at transfer 4 (the first sequenced time point) and increased in frequency over time (Table 2). The other two genes encoded hypothetical proteins that were deleted from the genome between timepoints 8 and 12 (Table 2). These genes are located next to each other and are near the several terminally redundant repeats EfV12-phi1 uses to circularly permute its genome, suggesting a likely mechanism for excision of these genes.

**Table 2:** All mutations present in phage EfV12-phi1 at the final timepoint.

Replicate	Condition	Gene/predicted function	Locus tag	Type	AA change	Frequency
1	Coevolution	Hypothetical protein 8	EFV12PHI1_123	Whole gene deletion	-	-375× coverage
1	Coevolution	Hypothetical protein 9	EFV12PHI1_126	Whole gene deletion	-	-545× coverage
1	Coevolution	Tail fiber	EFV12PHI1_98	Tandem duplication	-	+3× coverage
1	Coevolution	Capsid and scaffold	EFV12PHI1_97	Non-synonymous snp	N306K	99%
2	Coevolution	Hypothetical protein 8	EFV12PHI1_123	Whole gene deletion	-	-250× coverage
2	Coevolution	Hypothetical protein 9	EFV12PHI1_126	Whole gene deletion	-	-58× coverage
2	Coevolution	Tail fiber	EFV12PHI1_98	Tandem duplication	-	+5× coverage
2	Coevolution	Capsid and scaffold	EFV12PHI1_97	Non-synonymous snp	N306K	99%
3	Coevolution	Hypothetical protein 8	EFV12PHI1_123	Whole gene deletion	-	-33× coverage
3	Coevolution	Hypothetical protein 9	EFV12PHI1_126	Whole gene deletion	-	-30× coverage
3	Coevolution	Tail fiber	EFV12PHI1_98	Tandem duplication	-	+3× coverage
3	Coevolution	Capsid and scaffold	EFV12PHI1_97	Non-synonymous snp	N306K	77.5%
4	Coevolution	Hypothetical protein 8	EFV12PHI1_123	Whole gene deletion	-	-896× coverage
4	Coevolution	Hypothetical protein 9	EFV12PHI1_126	Whole gene deletion	-	-896× coverage
4	Coevolution	Tail fiber	EFV12PHI1_98	Tandem duplication	-	+5× coverage
4	Coevolution	Tail fiber	EFV12PHI1_98	Non-synonymous snp	R1460H	23.9%
4	Coevolution	Capsid and scaffold	EFV12PHI1_97	Non-synonymous snp	N306K	93.5%
1	Phage control	Capsid and scaffold	EFV12PHI1_97	Non-synonymous snp	N306K	81.3%
1	Phage control	Hypothetical protein 8	EFV12PHI1_123	Whole gene deletion	-	-5× coverage
1	Phage control	Hypothetical protein 9	EFV12PHI1_126	Whole gene deletion	-	-5× coverage
2	Phage control	Capsid and scaffold	EFV12PHI1_97	Non-synonymous snp	N306K	90.3%
2	Phage control	Hypothetical protein 8	EFV12PHI1_123	Whole gene deletion	-	-50× coverage
2	Phage control	Hypothetical protein 9	EFV12PHI1_126	Whole gene deletion	-	-48× coverage
3	Phage control	Capsid and scaffold	EFV12PHI1_97	Non-synonymous snp	N306K	92.1%
3	Phage control	Hypothetical protein 8	EFV12PHI1_123	Whole gene deletion	-	-14× coverage
3	Phage control	Hypothetical protein 9	EFV12PHI1_126	Whole gene deletion	-	-12× coverage
4	Phage control	Capsid and scaffold	EFV12PHI1_97	Non-synonymous snp	N306K	86.8%
4	Phage control	Hypothetical protein 8	EFV12PHI1_123	Whole gene deletion	-	-20× coverage
4	Phage control	Hypothetical protein 9	EFV12PHI1_126	Whole gene deletion	-	-18× coverage


*All phage mutations are from timepoint 16 in the coevolution and phage control populations. Frequencies given as population frequency for snps and fold coverage increases/decreases in the population for duplications and deletions. Locus tags correspond to GenBank record MH880817.*

**FIGURE 5:**
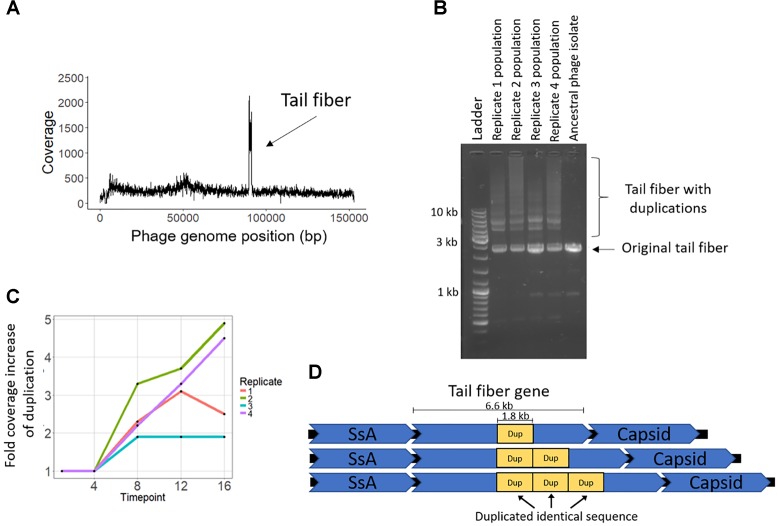
Phage EfV12-phi1 evolved tandem duplications in the tail fiber gene to increase its infectivity. **(A)** Average coverage along the phage genome for the phage population of replicate 2 at the final timepoint. Duplication was first noticed by this spike in sequencing coverage. Reads were mapped to the original phage genome so the duplication in the tail fiber appears as a spike in coverage. **(B)** Duplications in the tail fiber visualized by PCR using primers that flank the tail fiber. Duplications resulted in a larger amplicon. Each replicate population at the final timepoint is shown as well as the ancestral phage. **(C)** Presence of tail fiber duplications over time shown by the fold coverage increase in the duplicated region divided by the average coverage of the rest of the phage genome. **(D)** Schematic of the phage tail fiber tandem duplication within the gene. Reads spanning the tail fiber gene containing up to three duplications (four total copies of duplicated sequence) were seen with MinION long read sequencing.

The coevolution-specific phage mutation occurred in a gene encoding a putative tail fiber. Partial duplications of this gene occurred in all four coevolution replicates and never in the phage controls. Specifically, a 1.8 kb segment of a 6.6 kb putative tail fiber gene underwent in-frame tandem duplications (Figure 5). Over time, replicates acquired varying numbers of duplications 400 bp upstream of a predicted carbohydrate-binding domain. The duplication was initially observed as an increase in sequencing coverage present in all four populations beginning between transfers four and eight and persisting until the end of the experiment (Figure 5A,C). PCR was performed with primers were flanking the entire duplication so that the amplicon would increase in size if duplications occurred. For coevolved phage populations (Figure 5B) and isolates (data not shown), multiple amplicons of increasing size were observed that represent the size of the original tail fiber gene as well as larger tail fiber genes that contain duplications.

MinION long-read sequencing was performed on a phage isolate from the final timepoint of population 4 to resolve the duplication. Of the 1,021 reads spanning the entire tail fiber gene, 134 reads had no duplication (the original tail fiber gene), 852 reads had one duplication (two tandem copies of the duplicated sequence), 32 reads had two duplications, two reads had three duplications, and one read had four duplications. Thirty-four reads were found to consist of only tandem copies of the duplicated 1.8 kb sequence, ranging from 4 to 11 copies (7 to 20 kb in length). The mechanism by which these tandem duplications altered phage infectivity is currently not known; the duplication did not appear to be a diversity-generating mechanism, as only a single replicate acquired a SNP within the duplicated region (Table 2). The duplications were first detected at transfer eight, after a dramatic increase in bacterial abundance, which we attribute to the evolution of resistance to phage infection. The timing and exclusive occurrence in the coevolution treatment suggests that these tail fiber duplications were a phage response to the bacterial evolution of resistance.

## Discussion

To our knowledge, this represents the first effort to characterize phage-bacteria coevolution in *Enterococcus* – a common commensal in the gut microbiome that is also an important opportunistic pathogen. Similar to other well characterized systems, the experiments revealed coevolutionary arms race dynamics between *E. faecium* and its phage involving mutations in phage tail fibers and bacterial surface structures. They further demonstrated parallel coevolution among replicates and therefore predictable molecular adaptation. In particular, we identified bacterial exopolysaccharide mutations suggestive of hindering phage adsorption and RNA polymerase β’ subunit mutations with the potential to disrupt the phage replication cycle. However, we also identified what appears to be an unknown phage escape strategy involving large tandem repeats in the tail fiber gene. While some of the basic dynamics and molecular mechanisms of coevolution appear to be similar across many phage-host pairs (Labrie et al., 2010; Samson et al., 2013), experimental coevolution in this understudied system allowed us to quickly identify unique adaptation strategies.

Coevolving bacteria acquired mutations in the *yqwD2* gene, and we hypothesize that these mutations are at least partially responsible for the resistance phenotype seen in *E. faecium* coevolving with phage EfV12-phi1. The *yqwD2* gene is part of a capsule production operon that is well conserved among Firmicutes; it is known as Yqw in *Bacillus subtilis*, in *Streptococcus pneumoniae*, and Eps in *Streptococcus thermophilus* (Stingele et al., 1996; Bentley et al., 2006; Palmer et al., 2012). In *Streptococcus thermophilus*, the *epsD* gene (35% amino acid identity to *E. faecium yqwD2*) encodes a cytoplasmic tyrosine kinase that regulates the activity of EpsE, a phosphogalactosyltransferase. Disruption of either *epsD* or *epsE* abolished extracellular polysaccharide synthesis (Minic et al., 2007). Mutations in exopolysaccharide production genes have been shown to inhibit phage infection in *E. faecalis* (Teng et al., 2009) and *Lactococcus* *lactis* (Forde and Fitzgerald, 1999). Interestingly, two of these mutations occurred at residue 58 and one at residue 59 which are the beginning of a conserved nucleotide binding motif (GEGKS) (Stingele et al., 1996). A homologous protein structure within the conserved domain database (CDD) shows that this region of the protein is highly accessible. In line with protein models previously proposed (Stingele et al., 1996), perhaps these mutations interfere with the function of YqwD2, subsequently altering the structure, length, or quantity of exported exopolysaccharides (Bastin et al., 1993; Morona et al., 1995; Minic et al., 2007). While the phage receptor of phage EfV12-phi1 is unknown, distantly related phages *Staphylococcus* phage K and Bacillus phage SP01 bind to cell wall teichoic acids (Yasbin et al., 1976; Estrella et al., 2016). Similarly, phage EfV12-phi1 may bind to certain motifs of exopolysaccharides, so that modification of exopolysaccharides hinders phage adsorption. Further, several bacterial mutations that were not conserved among all replicates encoded sugar metabolism and modification functions which could also alter the structure and modifications present on exopolysaccharides. Future genetic knockout experiments will be useful in determining the degree to which these mutations confer resistance.

Coevolving bacteria also acquired mutations in the *rpoC* gene, which encodes the RNA polymerase β’ subunit. Phage EfV12-phi1 does not encode its own RNA polymerase, so it needs to interact with the host RNA polymerase both to shut down transcription of host genes and to transcribe phage genes. Mutations in the RNA polymerase *rpoC* gene could be a mechanism to resist phage infection by disrupting RNA polymerase activity. Phages produce proteins to bind or modify host RNA polymerase subunits, including the β’ subunit, to shut down host transcription and increase affinity for phage DNA (Mailhammer et al., 1975; Hesselbach and Nakada, 1977; Hodgson et al., 1985; Nechaev and Severinov, 2003). The mutation of residues that are modified or bound by phage proteins during infection could be a mechanism by which *E. faecium* can resist infection by phage EfV12-phi1. Five of the six different *rpoC* mutations observed were unique to single replicates, but all are located near each other in the 3D structure of RpoC, which suggests they all provide resistance to phage EfV12-phi1 through a common mechanism. The mutations in RpoC are localized similarly as the mutations that arise with the genetic suppressors of a protein, DksA that regulates *Escherichia coli* RpoC in response to nutrient availability (Rutherford et al., 2009). This suggests that the bacterial resistance arises through a general RpoC suppression mechanism that reduces phage success although it may not be driven by direct interaction with between phage proteins and RpoC.

The only phage mutations unique to coevolution (and not present in the evolution of phage EfV12-phi1 to naive host) were tandem duplications within a putative tail fiber gene. Myoviruses have short and long tail fibers, the latter of which are responsible for scanning the host cell surface and identifying the receptor. This gene has been confirmed to be the long tail fiber in a closely related phage, phiEF24C (Uchiyama et al., 2011). A point mutation in the homologous tail fiber of phiEF24C was seen to increase adsorption to several strains of *E. faecalis*. The duplication observed in this experiment occurs in a region of the gene that differs between EfV12-phi1 and phiEF24C. Protein homology analysis of the gene indicates a predicted carbohydrate-binding domain 100 nucleotides downstream from the duplicated region, but no conserved domains were predicted within the duplication itself. The duplicated region does not appear to generate sequence diversity which might allow recognition to different bacterial surface receptors, as has been observed in phage aaa (Meyer et al., 2012). The timing of EfV12-phi1 tandem duplications suggests that they are a response to the evolution of bacterial resistance to phage infection. Overall, we speculate that phage may respond to bacterial capsule changes through modifications in the tail fiber. Although mutations in the tail fibers are common mechanisms by which phages adapt to modified bacterial receptors (Tétart et al., 1996; Scanlan et al., 2011), examples of duplications as large as the one seen in this study (1.8 kb per duplication) have not been seen before.

Phage therapy has long been a proposed solution to the growing problem of antibiotic resistant bacteria, with recent successful cases of phage therapy in the United States following a compassionate use exemption. However, phage therapy is limited by a lack of well characterized phages infecting human pathogens (Duplessis et al., 2017; Schooley et al., 2017; Zhvania et al., 2017). Phage therapy utilizes phage cocktails, which include a mix of different phages with orthogonal targets to counter the evolution of bacterial resistance. Understanding the dynamics and outcomes of bacteria-phage interactions using experimental coevolution would facilitate phage cocktail design. For example, EfV12-phi1 has broad host-range and selects for *E. faecium* mutations related to exopolysaccharide synthesis, suggesting that a cocktail including EfV12-phi1 would be most effective if the other cocktail phages targeted host structures other than exopolysaccharide.

Phage EfV12-phi1 may have therapeutic potential, given that it is widespread and the host range was previously (Yoong et al., 2004) found to include a wide range of pathogens. Predictability of phage-host interactions is desirable to ensure safety of phages and for phage cocktail design. In this phage-host pair, we observed consistent outcomes from all four replicates, despite the stochasticity of mutations that lead to those outcomes. Nine of the eleven observed bacterial mutations were not shared among all replicates, but the functions encoded by these genes shared similar features (hydrolases, transferases, sugar metabolism/modification).

In these experiments, in just 8 days, we quickly identified phage and host genes that are under selection during coevolution. Experimental manipulation of phage-host interactions, and periodic tracking of their mutational trajectories, offers exceptional insight into the mutational arms race – beyond traditional sequencing and annotation efforts. While coevolution in artificial laboratory conditions may not be reflective of coevolution that happens in a natural environment, learning about the potential outcomes of coevolution provide useful information. As microbial culturing and enumeration becomes increasingly automated, a large number of phage-host interactions can be tested in order to thoroughly investigate the mechanism of phage-host co-evolution in a diversity of clinically relevant hosts. Such insights are critical to the eventual development of phage therapies for clinical use.

## Data Availability

All sequencing data has been deposited to the SRA at PRJNA490385. The genome for phage EfV12-phi1 can be found at GenBank: MH880817 and our assembly of *Enterococcus faecium* TX1330 can be found at GenBank assembly accession: GCA_003583905.1.

## Author Contributions

KW and JM conceptualized and designed the experiments. SW, TG, and CW performed the experiments. SW, AO, and WE did the sequencing analysis. SW, AO, KW, and JM wrote the paper. All authors edited the paper.

## Conflict of Interest Statement

The authors declare that the research was conducted in the absence of any commercial or financial relationships that could be construed as a potential conflict of interest.
