# Supplementary material for: Predictable Molecular Adaptation of Coevolving Enterococcus faecium and Lytic Phage EfV12-phi1
Source: Front Microbiol. 2019 Jan 31;9:3192. doi: 10.3389/fmicb.2018.03192 (PMC6365445; doi:10.3389/fmicb.2018.03192)
Supplement: Supplementary file 1 [file Table_1.DOCX]

**Table S1. Locus tags for genes mutating in *Enterococcus faecium* TX1330.**

| Gene | Locus tag GCA_000159675.1 | Locus tag GCA_003583905.1 |
| --- | --- | --- |
| RpoC | HMPREF0352_2730 | D3Y30_03855 |
| Histidinol-phosphatase (EC 3.1.3.15) CDS | HMPREF0352_0179 | D3Y30_13030 |
| *yqwD2* | HMPREF0352_1902 | D3Y30_11120 |
| Malonate decarboxylase beta subunit / Malonate decarboxylase gamma subunit CDS | HMPREF0352_0928 | D3Y30_01740 |
| Predicted hydrolase of the HAD Superfamily CDS | HMPREF0352_0295 | D3Y30_06415 |
| murA - UDP-N-acetylglucosamine 1-carboxyvinyltransferase | HMPREF0352_0323 | D3Y30_06555 |
| hydrolase, haloacid dehalogenase-like family CDS | HMPREF0352_1574 | D3Y30_13500 |
