# Supplementary material for: Predictable Molecular Adaptation of Coevolving Enterococcus faecium and Lytic Phage EfV12-phi1
Source: Front Microbiol. 2019 Jan 31;9:3192. doi: 10.3389/fmicb.2018.03192 (PMC6365445; doi:10.3389/fmicb.2018.03192)
Supplement: Supplementary file 2 [file Image_1.pdf]

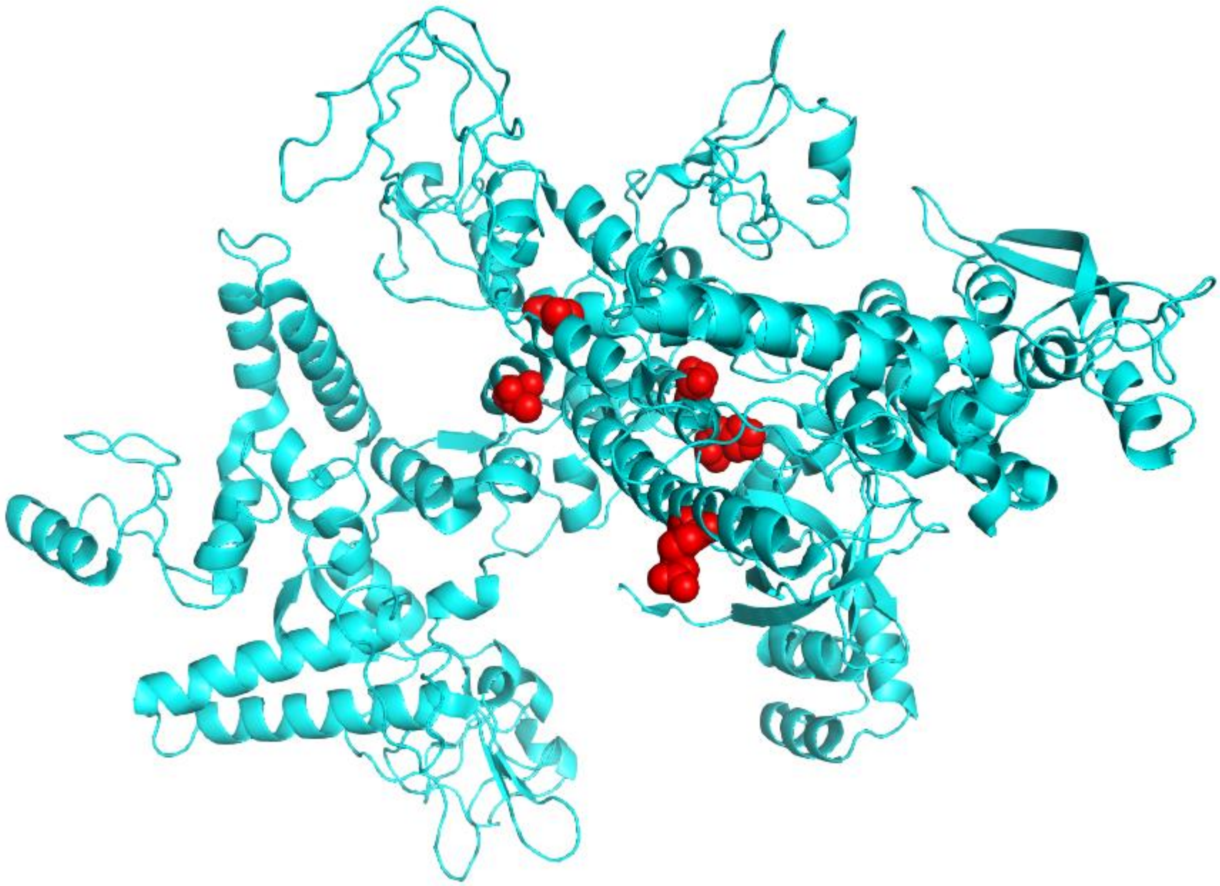

**Figure S1. Structural representation of RpoC with the positions of the mutations highlighted in red.**

Structure of *E. coli* RNA polymerase B’ shown with corresponding mutations in *E. faecium* TX1330 RNA polymerase B’ highlighted.
